# Expression and purification of hepatitis B surface antigen S from *Escherichia coli*; a new simple method

**DOI:** 10.1186/1756-0500-5-125

**Published:** 2012-03-01

**Authors:** Mohamed S Elghanam, Ahmed S Attia, Hussein A Shoeb, Abd Elgawad M Hashem

**Affiliations:** 1 Department of Microbiology & Immunology, Faculty of Pharmacy, Cairo University, Cairo, Egypt 11562

**Keywords:** Hepatitis B, HBsAg, Purification, GST-fusion

## Abstract

**Background:**

Hepatitis B is a liver disease primarily caused by hepatitis B virus (HBV) infection. It is distributed worldwide and associated with high mortality and morbidity rates. HBV infections can be avoided by the administration of the currently available vaccine and can be easily diagnosed through commercially available kits. Both the vaccine and the diagnostic kits depend on using the hepatitis B surface antigen (HBsAg) as an antigen. Developing countries such as, Egypt, suffer from the widespread of HBV infections and the limited resources to provide adequate supplies of either the vaccine or the diagnostic kits. Therefore the need for an easy, rapid, low cost method to produce HBsAg is urgently needed within this setting.

**Findings:**

To achieve this goal, the gene encoding the HBsAg(S) protein was cloned and expressed as a fusion protein with a GST tag in *Escherichia coli*. The recombinant protein was successfully expressed and purified in both good quality and quantity.

**Conclusions:**

The simplified and the relatively low cost of the used protocol make this an attractive alternative to protocols currently used for the purification of HBsAg(S). The exploiting of this achievement for new diagnostics can be directed for application in the developing countries where they are extremely needed.

## Background

Hepatitis B disease is a widely spread disease; it is estimated that approximately 2 billion people (one third of the world's population) have serological evidence of past or present HBV infection, and more than 350 million people are chronically infected [1]. It is highly endemic in developing regions with large population such as South East Asia, China, Sub-Saharan Africa and the Amazon basin, where at least 8% of the population are HBV chronic carriers [2].

The *env *gene of HBV codes for three related proteins: (1) S protein (HBsAg(S)), a 226 amino acids protein identified as a major protein constituent of the HBV envelope; (2) a 'middle' protein carrying 55 amino acids at the N-terminus encoded by the pre-S2 portion of the pre-S region; (3) and a 'large' protein encoded by the whole ORF (pre-S1, pre-S2 and S, 389aa). The latter 2 proteins represent the minor component of HBV envelop [3,4]. Among the three proteins, HBsAg(S) has the highest density of epitopes against HBsAb [5].

HBsAg has been used for a long time as a vaccine candidate and as a diagnostic immunoassay component. In early 1980s, plasma collected from chronic HBsAg carriers was used as a source of HBsAg. However, this came with bio safety concerns and required rigorous heat or chemical inactivation processes. However upon the development of recombinant DNA techniques HBsAg was expressed and purified in different systems both eukaryotic and prokaryotic one [6].

The methods that are currently used to purify HBsAg are either very costly, due to the requirement of using immunoaffinity purifications steps, or time consuming which would add also to the overall cost upon large scale application [7]. In developing countries (like Egypt), where financial resources are limited and HBV infections are prevalent [8,9], finding a rapid, low cost method for production of HBsAg is of an urgent need. In this study, we describe the expression and the purification of a recombinant HBsAg(S) as a GST fusion protein from *Escherichia coli *using a simple, low cost protocol.

## Materials and methods

### Bacterial strains, plasmids and growth condition

*Escherichia coli *strains DH5α, and DB3.1 were used for the cloning and expression experiments. *E. coli *was regularly grown in either Luria Broth (LB) or Nutrient Broth (NB) at 37°C with shaking at 180 rpm or on media solidified with 1.5% wt/vol agar. When appropriate the media were supplemented with ampicillin in a final concentration of 100 μg/ml or kanamycin in a final concentration of 25 μg/ml.

**Figure 1:**
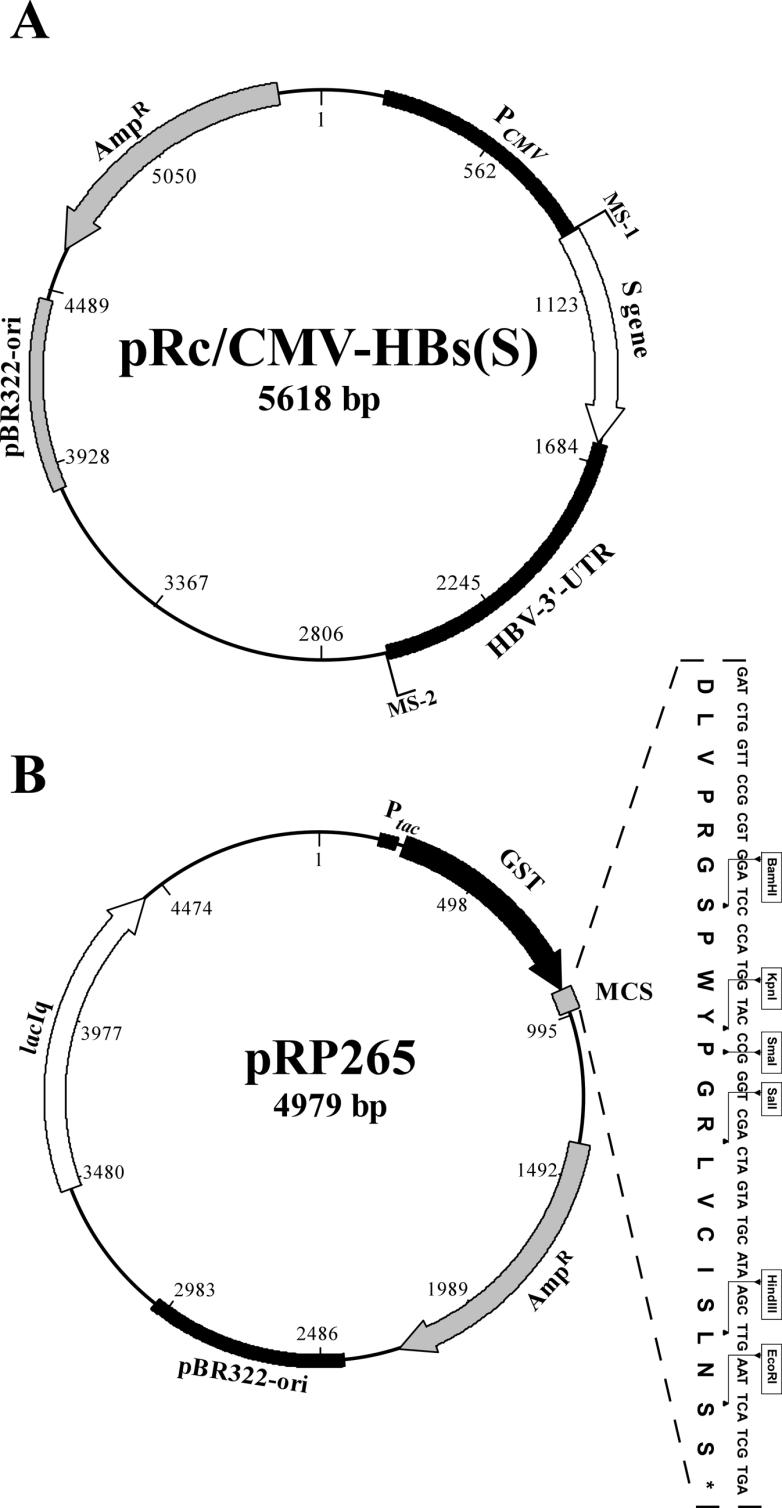
**Diagrammatic maps of the important plasmids used in this study**. (A) Map of the pRc/CMV-HBs(S) vector that is used as a source of the S gene of HBV. MS-1 & MS-2 indicate the positions of the primers used to amplify the S gene and the HBV-3'-UTR. (B) Diagrammatic map of the pRP265 vector used for cloning and expression the HBV S gene. The insert is showing the DNA and amino acid sequence of the multi cloning site (MCS) where the insert will be cloned. The maps in this figure were generated using the BioEdit software (Ibis Biosciences, USA).

The commercially available vector pRc/CMV-HBs(S) (Aldevron, USA) (Figure 1A) was used as a source for the S gene encoding the HBsAg(S). This vector is 5618 bp and carries the HBsAg(s) gene downstream of the CMV immediate-early promoter for expression in mammalian cells. In addition the plasmid carries the HBV-3'-UTR which was found essential for the production of protein in bacterial and mammalian cells. The selection marker on this vector is a β-lactamase gene that confers ampicillin resistant phenotype. For replication in prokaryotic cells, the plasmid contains the pBR322 origin of replication [10].

The expression vector pRP265 (NCCB, Netherlands) (Figure 1B) was chosen to express the gene encoding the S gene as a fusion protein in *E. coli*. This vector is a general cloning vector for recombinant protein production and it depends on cloning of the gene of interest in a polylinker site to form a fusion protein with Glutathione-s-transferase (GST) under the control of *tac *promoter. The *tac *promoter is a hybrid promoter derived from sequences of the *trp *and the *lac *UV5 promoters allowing more efficient controlled expression of foreign genes at high levels in *E. coli *[11]. It encodes Amp resistance gene as marker, LacI protein as inhibitor for the *tac *promoter and a polylinker sequence with the following restriction enzymes sites; HindIII, AccI, BamHI, EcoRI, KpnI, NcoI, NsiI, SalI, SmaI, and SpeI. The region between the gene encoding the GST-tag and the polylinker site contains a thrombin recognition site for subsequent removal of the GST-tag if needed. This vector replicates in *E*. *coli *through its pBR322 origin of replication.

Plasmid DNA was either purchased from the producing company or purified using QIAprep Miniprep Kit (Qiagen, USA) according to the manufacturer protocol.

### Primers design for cloning of the S gene

DNA primers for PCR amplification of S gene and the 3'-UTR were designed to clone the desired fragment in the KpnI and EcoRI sites of pRP265 due to the absence of these two sites within the sequence of desired piece of DNA. Primer MS-1 (5'-*tg*GGTACC*ac*ATGGAGAACATCACATCAGGAT-3', KpnI site underlined) contained the first 22 nucleotides of the S gene and two extra nucleotides were added between the translational start codon (ATG) and the KpnI site (underlined) in order to keep the S gene in frame with the gene encoding the GST-tag. Also two additional nucleotides were added upstream of the KpnI site to increase the efficiency of the cleavage by the restriction enzyme. Primer MS-2 (5'-*g*GAATTCCGAGATCCTCGCCGTCGGGCAT-3'; EcoRI site underlined) contained 22 nucleotides of the reverse stand of the end of the 3'-UTR of the HBV genome and an EcoRI restriction site (underlined) with an additional one nucleotide to increase the efficiency of the cleavage by the restriction enzyme.

### PCR amplification of the S gene

Primers MS-1 and MS-2 were used in a PCR reaction to amplify the S gene and the 3'-UTR using the vector pRc/CMV-HBs(S) as a template and the high fidelity DNA polymerase Ex-Taq (Takara, Japan) as a polymerase enzyme in a 50 μl volume reaction. Reaction mixtures were incubated in a programmable thermocylcer Touchgene gradient PCR (Touchgene Gradient, USA) using the following settings; initial denaturation: 2 min at 94°C, 30 cycles of (denaturation step: 50 seconds at 94°C, annealing step: 50 seconds at 50°C, and extension step: 2 min at 72°C) followed by final extension: 5 min at 72°C. PCR products were subjected to agarose gel electrophoresis in 0.8% wt/vol agarose gel and bands were visualized by staining with ethidium bromide and placing on a UV transilluminator.

### **Cloning of the S gene and the HBV-3**'**-UTR in rapid cloning vector pCR2.1**

In order to facilitate the restriction digestion and subsequent cloning of the amplified PCR product, 2 μl of the PCR product described above were ligated with 1 μl of the rapid cloning vector pCR2.1 (Invitrogen, USA) in the presence of 1 × ligation buffer and 3 units of T4 DNA ligase(promega, USA) at 16°C overnight. Three μls of the ligation mixture were then electroplated into 50 μl electrocompetent *E. coli *using Genepulser electroporator (Bio-Rad, USA) at 1.6 mV and 10 mm gap electroporator cuvette. Electroplated cells were recovered in 350 μl SOC media [12] for 60 min at 30°C with orbital shaking. Transformation mixtures were then plated on LB plates supplemented with 25 μg/ml kanamycin and is 20 μg/ml 5-bromo-4-chloro-3-indolyl-β-D-galactopyranoside (X-Gal). Plates were incubated at 37°C overnight and white colonies were then screened for the presence of the plasmid with the right insert using colony PCR using the primers pair AA437 (5'-AACAGCTATGACCATG-3') and AA438 (5'-GTAAAACGACGGCCAGT-3') that bind in the pCR2.1 vector backbone in the regions flanking the insert insertion site. The right plasmid was designated pCR2.1-HBsAg(S).

### **Sub-cloning of the S gene and the HBV-3**'**-UTR in pRP265**

The insert cloned in pCR2.1-HBsAg(S) as described above was digested out using the restriction enzymes KpnI and EcoRI (Promega, USA), gel purified, and ligated into the expression vector pRP265 that was restricted using the same enzymes, gel purified and treated with shrimp alkaline phosphatase (Promega, USA). The ligation mixtures were then electroporated into either *E. coli *DH5α or DB3.1. and plated on LB plates supplemented with ampicillin at a final concentration of 100 μg/ml. As a negative control restricted pRP265 without insert ligation was also transformed into *E. coli*. Ampicillin resistant *E. coli *were selected and screened for the presence of the right insert. The vector containing the right insert was designated pRP-HBsAg(S)-GST.

### Expression of the recombinant HBsAg(S) in *E. coli*

Cells carrying the pRP-HBsAg(S)-GST vector were grown overnight in NB/Amp, containing 2% (wt/vol) glucose to prevent any leaky expression of the recombinant protein, with shaking at 37°C. In the next morning, cells were sub-cultured in 50 ml of fresh medium through 1:100 dilution and allowed to grow to mid-logarithmic phase (~ 3 hours) at 37°C with shaking. When the cultures reached an OD_600 _~ 0.4, the expression of the recombinant protein was induced by the addition of Isopropyl β-D-1-thiogalactopyranoside (IPTG) in a final concentration of 1 μM. Induction was continued for different time periods (0, 3, 6, and 24 hours) and at different temperatures (30°C and 37°C) with shaking at 180 rpm. Aliquots were removed at different time points for analysis using SDS-PAGE using standard protocols [12].

### Solubilization of the inclusion bodies containing the recombinant HBsAg(S)

In order to check if the recombinant HBsAg(S) is included inside inclusion bodies a solubilization step was performed. Briefly, induced bacterial cultures were centrifuged at 3000 × g for 5 min at 4°C, pellets were re-suspended in 1 ml lysis buffer (50 mM Tris HCl (pH 8), 10% glycerol, and 0.1% Triton X-100) and sonicated on ice using a sonicator equipped with a microtip. Sonication was carried out on ice for six 10 s bursts at 200-300 W with a 10 s cooling period between each burst. The cell debris and the potential inclusion bodies were collected by centrifugation for 15 min at 10,000 × g at 4°C. The pellets were re-suspended into 1 volume of solubilization buffer I (50 mM Tris HCl (pH 8.0), 1 mM EDTA, 100 mM NaCl, and 8 M urea), and incubated for 60 min at room temperature. Then 9 volumes of solubilization Buffer II (50 mM KH_2_PO_4 _(pH 10.7), 1 mM EDTA, and 50 mM NaCl) were added and the mixture was incubated for additional 30 min at room temperature. After solubilization, the pH was re-adjusted to 8 with HCl and incubated for 30 min at room temperature. The un-dissolved inclusion bodies and cell debris were collected by centrifugation for 5 min at 10,000 × g at 4°C. The pellets were discarded and the supernatant containing the solubilized inclusion bodies and the recombinant HBsAg(S) was removed for SDS-PAGE analysis and protein purification.

### Refolding of the recombinant HBsAg(S)

In order to be able to purify the recombinant HBsAg(S) released from the solubilized inclusion bodies, it needed to be refolded so it can bind to the GST beads for purification. The refolding of the protein needed dialysis against dialysis buffers (50 mM glycine, 10% glycerol, 5 mM mercaptoethanol, 50 mM Tris HCl (pH 8), 50 mM NaCl, and 1 mM EDTA) containing decreasing concentrations of urea (4 M, 2 M,1 M, and 0 M) [12]. A final dialysis run was carried out against the refolding buffer diluted 1:3 with distilled water. The equilibrated protein was then ready for affinity purification.

### Purification of the recombinant HBsAg(S)

Dialyzed protein was mixed with 1/10 volume glutathione sepharose beads slurry (Pierce, USA) with gentle shaking for 1 h at room temperature. The mixture was then packed into column and the flow through was allowed to pass out of the column that was later washed with five volumes PBS. To elute the bound protein, aliquots of a low pH elution buffer (0.1 M glycine HCl, pH 2.7) were applied to the column and the collected fractions were rapidly neutralized using 1/10 volume neutralization buffer (2 M Tris, pH 8.0). Fractions collected were analyzed qualitatively by measuring absorbance at 280 nm, and small aliquots of the fractions with high readings were subjected to electrophoresis in 15% SDS-PAGE gel and stained with Coomassie blue for visualization. The total volume of the purified protein solution was adjusted to 1.5 ml and quantitative determination of the purified protein was determined using the Bradford assay [12].

### Statistical analyses

Statistical analyses were carried out by applying either the Student *t test *or analysis of variance (ANOVA) using GraphPad Prism software (GraphPad Software, Inc., USA) where *p *values < 0.05 were considered statistically significant.

## Results

### PCR amplification and cloning of the S gene

**Figure 2:**
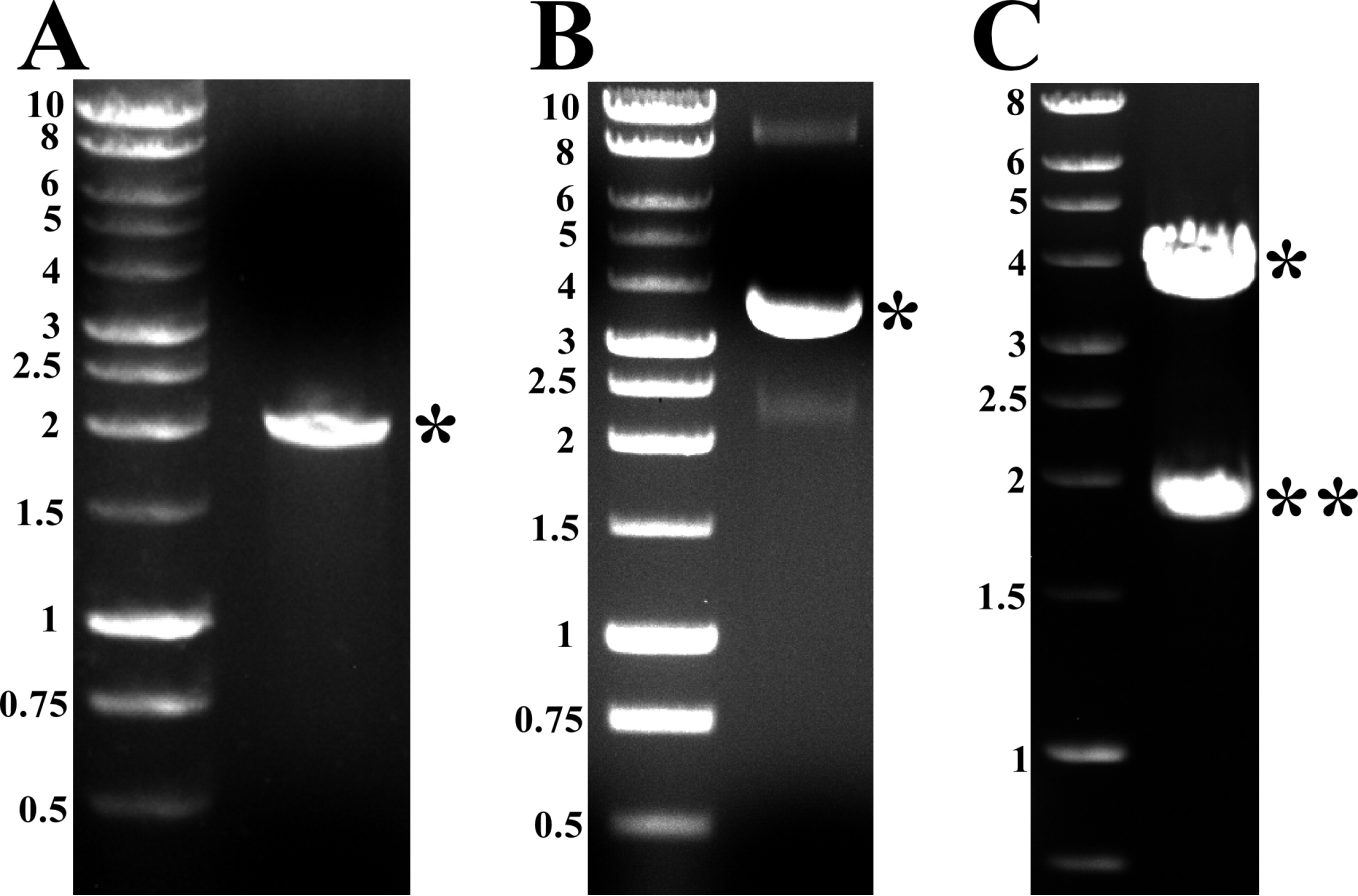
**Cloning of the HBV S gene and 3'-UTR in the rapid cloning vector pCR2.1**. (A) An image of an agarose gel showing the MS-1-MS-2 PCR product. The * indicates the position of the PCR product. (B) An image of an agarose gel showing the supercoiled pCR2.1-HBsAg(S) and the * indicates the position of the plasmid. (C) An image of an agarose gel showing the KpnI/EcoRI digestion products of pCR2.1-HBsAg(S). The * indicates the position of the linearized plasmid and the ** indicates the position of the dropped insert. The left lane in each gel contains 1 kb DNA ladder (Promega, USA); the sizes of the respective bands is indicated on the left side of each panel.

**Figure 3:**
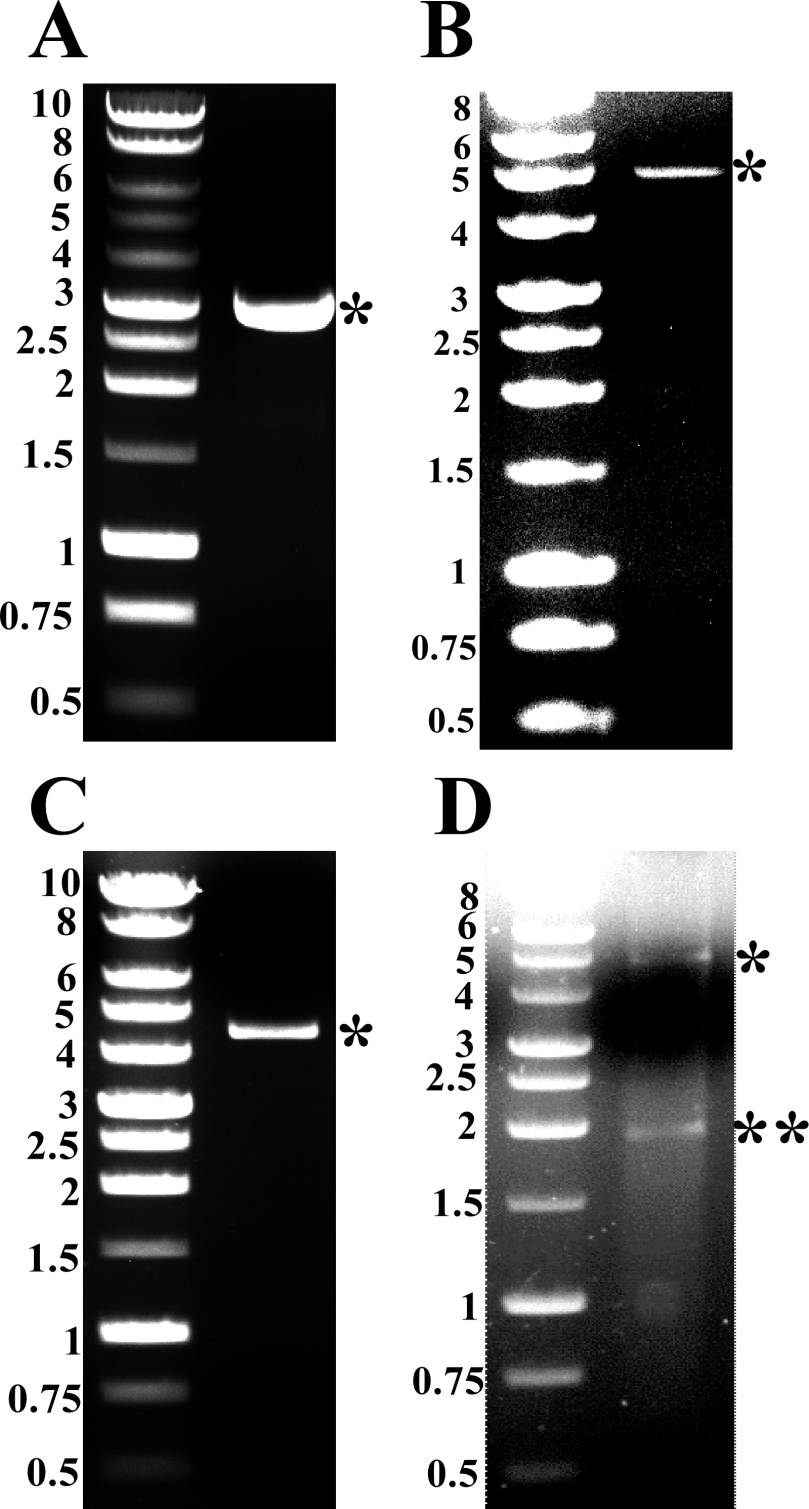
**Sub-cloning of the HBV S gene and 3'-UTR in the expression vector pRP265**. (A) An image of an agarose gel showing the supercoiled pRP265 and the * indicates the position of the plasmid. (B) An image of an agarose gel showing the KpnI/EcoRI digestion products of pRP265 and the * indicates the position of the linearized plasmid. (C) An image of an agarose gel showing the supercoiled pRP-HBsAg(S)-GST and the * indicates the position of the plasmid. (D) An image of an agarose gel showing the KpnI/EcoRI digestion products of pRP-HBsAg(S)-GST. The * indicates the position of the linearized plasmid and the ** indicates the position of the dropped insert. The left lane in each gel contains 1 kb DNA ladder (Promega, USA); the sizes of the respective bands is indicated on the left side of each panel.

DNA primers MS-1 and MS-2 were used to amplify the S gene of HBV together with the HBV-3'-UTR using the pRc/CMV-HBs(S) as a template. The PCR reaction produced a single strong band of the expected size (i.e. ~ 2 kb) as shown in Figure 2A. The produced PCR product was cloned into the rapid ligation vector pCR2.1 to produce the vector pCR2.1-HBsAg(S) (Figure 2B). Restriction digestion of pCR2.1-HBsAg(S) with EcoRI and KpnI resulted in the linearization of the pCR2.1 vector and the release of the S gene-HBV-3'-UTR fragment (Figure 2C). The later fragment was then gel purified and ligated into the expression vector pRP265 that has been digested with KpnI and EcoRI (Figure 3A &3B). The resultant plasmid pRP-HBsAg(S)-GST (Figure 3C) was digested with EcoRI and KpnI producing ~ 5 kb band corresponding to linearized pRP265 vector and ~ 2 kb band corresponding to the S gene-HBV-3'-UTR insert (Figure 3D)

### Expression and purification of HBsAg(S)-GST from *E. coli*

**Figure 4:**
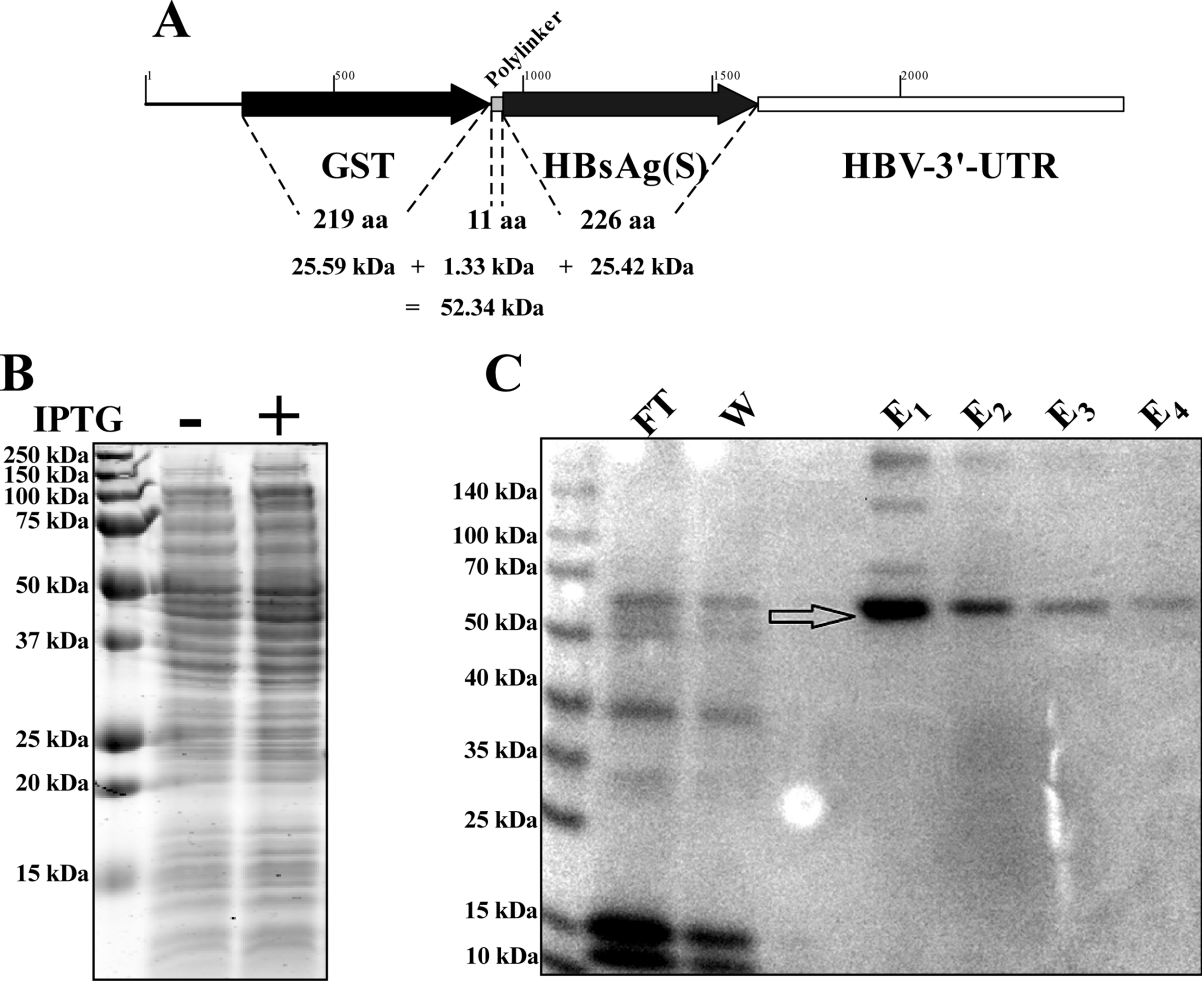
**Expression and purification of the HBsAg(S)-GST fusion protein**. (A) Schematic diagram showing the DNA region cloned in pRP-HBsAg(S)-GST and the sizes of the predicted encoded protein. The map in this figure was generated using the BioEdit software (Ibis Biosciences, USA). (B) An image of a Coomassie blue-stained SDS-PAGE gel of cell lysates of *E. coli *strains carrying the pRP-HBsAg(S)-GST that were either not induced with IPTG (-) or induced with 1 mM IPTG for 3 hours (+). The first lane contained protein molecular weight marker (BioRad, USA) and the sizes of the respective bands is indicated on the left side of the panel. (C) An image of a Coomassie blue-stained SDS-PAGE gel of purified HBsAg(S)-GST fusion protein. The first lane contained protein molecular weight marker (Fermentas, USA) and the sizes of the respective bands is indicated on the left side of the panel. Lane 2 contained a sample of the column flow through (FT), lane 3 contained a sample of the column wash (W), while lanes 5-8 contain samples of the fractions eluted from the GST column. The arrow indicates the position of the main component eluted from the column with a band size of approximately 52 kDa.

The fusion protein that is encoded by the vector pRP-HBsAg(S)-GST (Figure 4A) consists of; (i) GST tag that contains 219 amino acids (aa) with a predicted molecular weight of 25.59 kDa, (ii) polylinker consisting of 11 aa with a predicted molecular weight of 1.33 kDa, and (iii) HBsAg(S) consisting of 226 aa with a predicted molecular weight of 25.42 kDa. Adding the three fragments makes the predicted molecular weight of the fusion protein ~52.34 kDa.

*E. coli *strain harboring the vector pRP-HBsAg(S)-GST was induced using IPTG to over-express the recombinant fusion protein HBsAg(S)-GST. Induction of the cells was followed by cell lysis through sonication, however upon running equivalent aliquots of the cytoplasmic lysates of both induced and un-induced cultures in SDS-PAGE gel did not show any increase in the abundance of a band around the expected size of the HBsAg(S)-GST (~52 kDa) (Figure 4B). This meant that the fusion protein HBsAg(S)-GST might not have been expressed in these cells. However, it was observed that the cells carrying pRP-HBsAg(S)-GST showed a lag in growth in liquid culture when compared to the cells carrying the empty vector pRP265 and their colonies were more sticky (data not shown). These observations suggested that pRP-HBsAg(S)-GST is expressed inside the cells, most probably in high concentrations, and the reason that the protein is not detected in the cytoplasmic lysates is that it might be included in inclusion bodies.

Inclusion bodies solubilization, followed by refolding then affinity chromatography purification resulted in the detection of the recombinant fusion protein HBsAg(S)-GST. A band corresponding to the predicted size (~52 kDa) was obviously detected in the eluted proteins from the GST column (Figure 4C). The detection of a single prominent band in the SDS-PAGE gel indicates that the purified protein is of high quality.

### Effect of induction time and temperature on the protein yield

**Figure 5:**
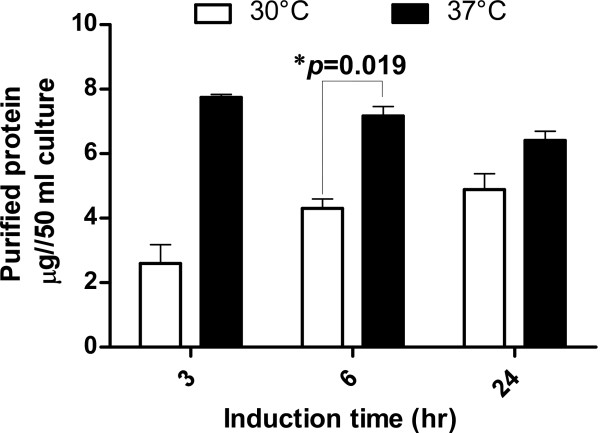
**Effect of induction time and temperature on the protein yield**. The amounts of the protein yield when the bacterial cultures were induced for 3, 6, and 24 hours either at 30°C (open bars) or 37°C (black bars) as determined using Bradford assay. The data presented is the average of the two independent experiments each one was done in triplicates and the error bars represent the standard error. The * indicates that the difference is statically significant with the indicated *p-*value as determined using the student *t*-test.

Bacterial cultures of the cell carrying the expression vector pRP-HBsAg(S)-GST were induced for different time intervals (3, 6, and 24 hours) and at different temperatures (30 and 37°C). At each time point, the recombinant protein was purified as described in the Materials and Methods section and the protein content was assayed using Bradford assay. When the cultures were induced at 30°C, there was a gradual increase in the amount of the purified protein however, the differences between the three time points were statistically non-significant (*p *value > 0.05) (Figure 5, white bars). On the other hand, at 37°C, the highest amount of protein was obtained after 3 hours of induction then there was slight decrease in the recovered protein, but again the differences between the three time points were statistically non-significant (*p *value > 0.05) (Figure 5, black bars). The only significant difference was detected when comparing the amounts of the protein recovered after 6 hrs where the protein recovered at 37°C was significantly higher than that recovered after the same induction time at 30°C with *p *value 0.019. Upon analyzing the two variables (induction time and temperature) together via ANOVA, only the temperature factor showed a significant difference (*p *value < 0.0001). Extrapolating the obtained yields in this small scale experiment, the method described here will result in a protein yield of approximately 140 μg protein per liter of culture.

## Discussion

HBV infections are spreading worldwide and affecting a substantial proportion of the world's population [1,2]. The need for rapid and low cost reagents that can be used for the detection and prevention of such infections is highly urgent especially in the developing world where healthcare resources are evidently scarce [13-15].

HBsAg is one of the major antigens of HBV that constitutes a fundamental component in both diagnostic and preventive tools for HBV. Initially the source for such antigen was the plasma of high titer HBV carriers however this approach suffered from safety issues in addition to over-elaborate preparation process as well as high cost of preparation.

Cloning and expression of HBsAg components have been attempted many times. Attempts were carried on using the yeast cells *Saccharomyces cerevisiae *as a host [16,17] or in prokaryotic cells such as *E. coli *[18,19]. However, in all these early studies high levels of HBsAg expression were not reported [20]. Going back and forth between eukaryotic and prokaryotic systems several groups reported success in the expression of HBsAg with different advantages and disadvantages.

Expression in eukaryotic systems such as *Saccharomyces cerevisiae*, *Pichia pastoris *and insect expression systems results in HBsAg proteins that are glycosylated in different patterns and this glycosylation can affect the immunoreactivity of the product [5]. In this respect the *Pichia pastoris *is better than *Saccharomyces cerevisiae *in that proteins synthesized by *Pichia pastoris *may resemble the glycoprotein structure of higher eukaryotes [21] as the length of the oligosaccharide chains added post-translationally to proteins in *Pichia *is much shorter than that in *Saccharomyces cerevisiae *[22]. Other advantages of a system such as *Pichia pastoris *are the high level and stability of the protein expression and easy manipulation [5].

Prokaryotic systems in general and *E. coli *in particular, have the great advantages of low cost, easy manipulation, high level of expression of recombinant proteins. With the *E. coli *being capable of achieving about five times typical specific product formation rate [mg·g^-1^·h^-1^] of recombinant proteins that are expressed cytoplasmically as compared to *Pichia pastoris *and about 25% more increase in volumetric productivity [mg·L^1^·h^1^] [23]. In addition, there is a high rate of development in metabolic engineering strategies and tools that are looking into approaches to reveal metabolic bottle-necks in prokaryotic host cells. This progress makes a potentially sweet future for glycoengineering in *E. coli *for the production of human therapeutic drugs [24].

The purification process of the HBsAg from *Saccharomyces cerevisiae *described by Kobayashi *et. al*. [7] is long, complicated, and costly due to the use of immunoaffinity chromatography using antibodies for hepatitis B surface antigen bound to sepharose. Using fusion proteins such as poly histidine or GST followed by metal ion affinity chromatography promotes efficient recovery and purity of recombinant protein from crude cell extracts [5]. Different fragments of the HBsAg complex have been purified as a fusion protein with β-galactosidase [19], β-lactamase [25], or hexa-histidine [5]. To the best of our knowledge this is the first report of the expression of a GST fusion of the HBsAg(S) fragment which represents the major antigen of HBV. Using GST as a fusion tag has several advantages such as providing a higher degree of purification in a single chromatographic step, increased solubility of the recombinant protein, non-interference with the structure and function of the recombinant protein, providing immunogenic as well as biochemical assay of the recombinant protein, easy elution steps, and ease of cleavage and removal of the affinity tag [26].

The fusion protein was expressed in *E. coli *and despite the fact that GST usually increases the solubility of the fusion protein; the GST-HBsAg(S) fusion was included into inclusion bodies rather than being free in the cytoplasm. This required an extra step of inclusion bodies solubilization followed by dialysis to refold the recombinant protein. Despite this the protocol described here is simpler, faster, costs less than the one described by Kobayashi *et. al*, [7]. In addition the refolding can be achieved using other protocols as those described [12] leading to more time savings.

The protein yields obtained in the small scale experiments described in this study are very encouraging that high yields could be obtained upon applying large scale production. Determination of the immunogenicity (as vaccine candidate) or the sensitivity, specificity, accuracy and linearity (as a diagnostic immunoassay component) of the purified GST-HBsAg(S) is still required to prove the usefulness of the application of this product in developing countries such as Egypt.

## Conclusions

The construct and the method described in this study represent an attractive alternative to previously described protocols for the production and purification of HBsAg especially for countries of limited resources. The described protocol is characterized with the simplicity, low cost and high productivity.

## Abbreviations

HBV: Hepatitis B virus; HBsAg: Hepatitis B surface Antigen; PCR: Polymerase chain reaction; X-Gal: 5-bromo-4-chloro-3-indolyl-β-D-galactopyranoside; IPTG: Isopropyl β-D-1-thiogalactopyranoside.

## Competing interests

The authors declare that they have no competing interests.

## Authors' contributions

Both MSE and ASA carried out the experimental procedures. ASA drafted the manuscript. HAS and AMH designed and supervised the study. All authors (except the deceased HAS) read and approved the final manuscript.
